# Beyond Biomarkers: Reclaiming the Central Role of Clinical Neurophysiology in Neuromuscular Disorders

**DOI:** 10.3390/brainsci16090999

**Published:** 2026-09-21

**Authors:** Marilena Mangiardi

**Affiliations:** 1 Neuroscience Department, San Camillo Forlanini Hospital, 00152 Rome, Italy; marilenamangiardi@gmail.com; 2 Neurophysiology Division, University of Pittsburgh Medical Center (UPMC) Salvator Mundi International Hospital, 00152 Rome, Italy

**Keywords:** clinical neurophysiology, electromyography, nerve conduction studies, biomarkers, neuromuscular disorders, precision medicine, electrophysiology

## Abstract

**Highlights:**

**What are the main findings?**

- Biological biomarkers and clinical neurophysiology provide complementary information on disease mechanisms and their functional expression.
- Across major neuromuscular disorders, neurophysiological assessment characterizes dynamic functional processes that may not be captured by biological or structural biomarkers alone.

**What are the implications of the main findings?**

- Integrating neurophysiological, molecular, imaging, and quantitative approaches may improve diagnosis, longitudinal assessment, and therapeutic evaluation.
- Artificial intelligence may enhance quantitative signal analysis, but its clinical implementation requires rigorous validation, physiological interpretation, and expert oversight.

**Abstract:**

Over the past two decades, advances in molecular genetics, immunology, neuroimaging, fluid biomarkers, and artificial intelligence have substantially transformed the diagnosis, classification, and treatment of neuromuscular disorders. However, increasing biological precision has also highlighted an important distinction between identifying disease mechanisms and characterizing their functional expression. Clinical neurophysiology occupies a specific position within this evolving diagnostic landscape by directly assessing nervous system function and providing objective information on impulse conduction, neuromuscular transmission, motor unit integrity, physiological reserve, and disease activity. Consequently, neurophysiological assessment contributes not only to diagnosis but also to differential diagnosis, longitudinal monitoring, prognostic assessment, and therapeutic evaluation. In this critical review, I examine the factors that have progressively shifted clinical neurophysiology from an interpretative clinical discipline toward a more procedural role. Using representative examples from myasthenia gravis, immune-mediated neuropathies, myopathies, and amyotrophic lateral sclerosis, I discuss how physiological information complements molecular, serological, imaging, and other biological biomarkers. I further examine the emerging role of artificial intelligence, including its potential for automated signal analysis and quantitative assessment, as well as current limitations related to generalizability, validation, and overreliance on pattern recognition. I propose a complementary conceptual framework in which biological and molecular approaches characterize disease mechanisms, while clinical neurophysiology defines their functional expression and clinical relevance in the individual patient. Within this framework, clinical neurophysiology should not be considered an alternative to precision medicine, but an integrated physiological dimension of it, connecting biological characterization with clinically actionable functional information.

## 1. Introduction

### From Queen of Neurology to Invisible Consultant

Modern neuromuscular medicine has undergone substantial transformation over the past two decades. Advances in molecular genetics, immunology, neuroimaging, fluid biomarkers, and targeted therapies have changed the way neuromuscular disorders are diagnosed, classified, and treated. Increasingly sophisticated diagnostic tools can identify disease mechanisms and biological subtypes with growing accuracy, enabling therapies directed toward specific molecular and immunological targets. Yet, while biological characterization has advanced considerably, an equally important dimension of neurological diagnosis may receive comparatively less attention: the physiological expression of disease. Identifying the mechanism responsible for a neurological disorder is not necessarily equivalent to understanding how that mechanism alters the function of the living nervous system. Disease mechanisms and disease expression therefore represent complementary, but not interchangeable, dimensions of clinical reasoning. Clinical neurophysiology has historically occupied this interface [1]. Unlike approaches primarily designed to identify structural or molecular abnormalities, electrophysiological investigations directly assess the functional consequences of disease in vivo. Nerve conduction studies and needle electromyography (EMG) became extensions of the neurological examination, allowing clinicians to localize lesions, distinguish pathophysiological mechanisms, characterize functional impairment, and refine differential diagnosis [2,3,4]. Their role therefore extended beyond diagnostic confirmation: neurophysiological findings contributed to the interpretation of clinical uncertainty and to therapeutic decision-making. The diagnostic landscape has nevertheless changed substantially. Disease-specific autoantibodies, next-generation sequencing, advanced imaging, fluid biomarkers, and, increasingly, artificial intelligence have reshaped diagnostic pathways across neuromuscular medicine. These advances have accelerated diagnosis, improved disease classification, and expanded therapeutic opportunities. At the same time, biological or structural abnormalities do not invariably indicate their degree of functional expression, clinical relevance, or activity in an individual patient. This distinction creates an important interface between increasingly detailed biological characterization and the functional information required for clinical decision-making.

**Figure 1:**
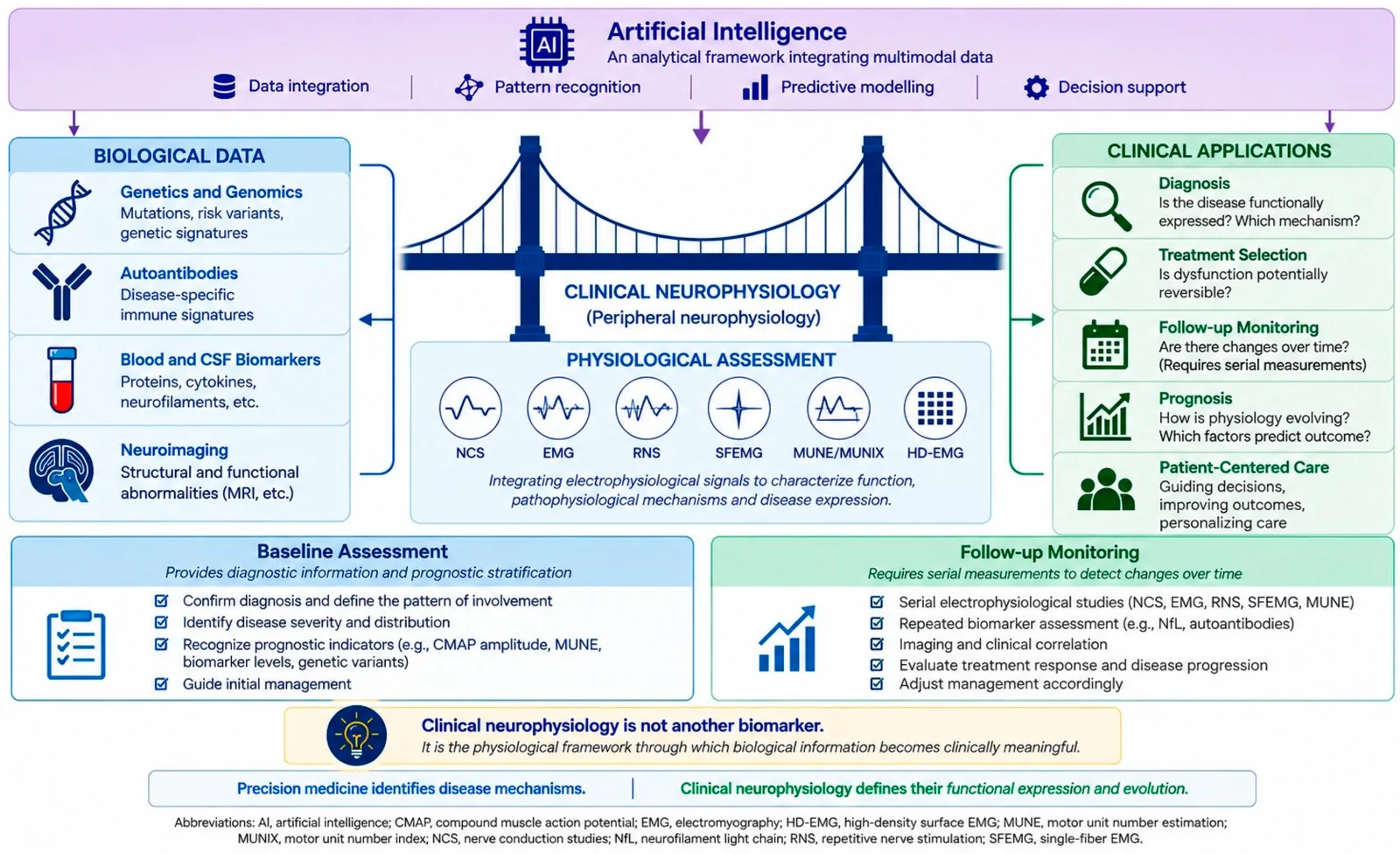
Clinical Neurophysiology: The Bridge Between Biological Precision and Clinical Decision-Making. Conceptual framework illustrating the role of peripheral clinical neurophysiology as a functional bridge between biological disease characterization and clinical decision-making in neuromuscular disorders. Genetics and genomics, autoantibodies, blood and cerebrospinal fluid biomarkers, and neuroimaging provide complementary molecular, immunological, biochemical, and structural information, whereas peripheral clinical neurophysiology directly assesses the functional expression of disease through nerve conduction studies (NCS), electromyography (EMG), repetitive nerve stimulation (RNS), single-fiber electromyography (SFEMG), motor unit number estimation (MUNE), motor unit number index (MUNIX), and high-density surface EMG (HD-sEMG). Artificial intelligence (AI) is represented as an overarching analytical framework capable of processing and integrating multimodal data rather than as an independent diagnostic modality. Baseline neurophysiological assessment may contribute to diagnosis, disease characterization, and prognostic stratification, whereas longitudinal follow-up requires serial measurements to identify physiological changes over time and assess disease progression and treatment response. Together, multimodal biological information and functional neurophysiological assessment may support more individualized clinical decision-making and patient-centered care.

Previous reviews, consensus documents, and methodological studies have established the diagnostic value of electrodiagnostic testing and highlighted the emergence of quantitative neurophysiological biomarkers in neuromuscular disease [5,6]. The purpose of the present review is therefore not simply to reaffirm or preserve the traditional diagnostic role of clinical neurophysiology. Rather, I critically examine how its role should be redefined within a diagnostic environment increasingly shaped by molecular biomarkers, genetics, imaging, and artificial intelligence. I propose that clinical neurophysiology provides physiological characterization complementary to biological characterization and that neurophysiological abnormalities may be interpreted as dynamic functional biomarkers linking disease mechanisms to phenotype, disease activity, and clinical decision-making. Through representative examples from myasthenia gravis (MG), immune-mediated neuropathies, myopathies, and amyotrophic lateral sclerosis (ALS), this review examines the clinical implications of this framework, its limitations, and its potential relevance to the future integration of biological and functional information in neuromuscular medicine (Figure 1).

## 2. Review Approach

This article was designed as a critical narrative review aimed at examining the evolving relationship between biological characterization and physiological assessment in contemporary neuromuscular disorders. Rather than providing an exhaustive systematic synthesis of the literature, the review was structured around representative clinical and conceptual questions relevant to the role of clinical neurophysiology in current diagnostic and therapeutic pathways. The literature supporting the present review was identified through a targeted search of the biomedical literature, with particular attention to studies addressing the diagnostic, pathophysiological, prognostic, and longitudinal contribution of clinical neurophysiology in neuromuscular disorders. Priority was given to international guidelines and consensus statements, landmark studies that contributed to the development of major neurophysiological concepts, and recent peer-reviewed original studies and reviews relevant to the interaction between electrophysiological findings, molecular and serological biomarkers, imaging, and emerging computational approaches. References were selected according to their relevance to the specific clinical and conceptual questions discussed rather than according to predefined criteria for systematic evidence synthesis. MG, immune-mediated neuropathies, myopathies, and ALS were selected as representative disease models because they illustrate different relationships between biological disease characterization and functional physiological assessment. Particular emphasis was placed on clinical scenarios in which these approaches provide complementary or potentially discordant information, including seronegative disease, conduction block and reversible conduction failure, active versus chronic muscle involvement, denervation and reinnervation, and longitudinal assessment of motor unit function. The literature concerning artificial intelligence was additionally selected to address both emerging applications in neurophysiological signal analysis and current methodological and implementation limitations. Accordingly, the literature selection was intended to support a critical conceptual synthesis of the available evidence rather than a quantitative comparison of diagnostic performance or a comprehensive systematic assessment of all published studies.

## 3. The Paradox of Modern Neuromuscular Diagnosis

The changing position of clinical neurophysiology in neuromuscular medicine reflects a conceptual rather than technological transition. Electrophysiological techniques remain standardized, quantitatively robust, and embedded in diagnostic criteria, yet diagnostic reasoning has increasingly shifted toward biological classification. The apparent paradox is therefore not technological but conceptual. As neuromuscular medicine has become progressively more successful in identifying the biological basis of disease, diagnostic reasoning has gradually shifted from physiological interpretation toward biological classification. This evolution has improved diagnostic accuracy and therapeutic stratification, while progressively repositioning neurophysiology from an exploratory discipline toward a more frequently confirmatory role. The following sections examine the implications of this transition.

### 3.1. Biological Precision Does Not Necessarily Mean Functional Understanding

Biomarkers have transformed neuromuscular medicine by enabling earlier diagnosis, improved disease stratification, and increasingly targeted therapies. Molecular genetics, disease-specific autoantibodies, advanced imaging, and fluid biomarkers can now identify biological mechanisms with a degree of specificity that was previously unattainable [7,8,9]. However, biological identification and functional characterization address different clinical questions. The distinction becomes particularly relevant when biological abnormalities and physiological dysfunction diverge. A disease-specific antibody may establish an immunological substrate without quantifying the resulting functional impairment; a pathogenic genetic variant may define disease susceptibility without indicating the timing or severity of functional expression; and biomarkers of neuroaxonal injury may reflect tissue damage without determining whether the associated deficit is physiologically reversible. Biological abnormalities therefore cannot be assumed to correspond directly to disease activity or functional status. Clinical neurophysiology addresses this gap by interrogating the functional consequences of disease in vivo [5,6]. Electrophysiological assessment can determine whether impulse conduction is preserved, neuromuscular transmission is impaired, motor units remain functionally viable, or dysfunction has progressed toward irreversible structural loss. Its contribution is therefore complementary to biological characterization: biomarkers help define the underlying disease process, whereas neurophysiology characterizes its functional expression and potential clinical relevance in the individual patient.

### 3.2. From Clinical Reasoning to Diagnostic Outsourcing

Electrodiagnostic evaluation has traditionally been conceived as an extension of the clinical examination and as an electrodiagnostic consultation, in which study design and interpretation evolve according to the clinical context and to the physiological findings obtained during the examination [5,10]. Importantly, its diagnostic contribution may extend beyond confirmation of the referring hypothesis: in patients evaluated for suspected polyneuropathy, electrodiagnostic consultation was confirmatory in only 39% of cases, while it changed the diagnosis or identified an additional diagnosis in 43% [11]. These observations emphasize the exploratory and interpretative potential of neurophysiological assessment. The expansion of molecular diagnostics has changed not only the information available to clinicians but also the sequence in which diagnostic questions are formulated. Electrophysiological investigations may increasingly be requested after a diagnostic hypothesis has already been established through clinical, imaging, serological, or genetic information, rather than during its initial construction. Although systematic evidence quantifying this shift remains limited, it raises an important conceptual question regarding the changing role of neurophysiology within contemporary diagnostic pathways. This shift may change the question addressed to the neurophysiologist. An exploratory consultation asks where dysfunction originates, which physiological mechanism best explains the phenotype, and whether alternative diagnoses should be considered. A confirmatory consultation instead asks whether predefined electrophysiological criteria are fulfilled or whether an established diagnostic hypothesis is supported. Both approaches are clinically legitimate, but an exclusively confirmatory model may restrict the interpretative contribution of neurophysiology and reduce a physiological consultation to verification of an already established diagnostic framework. I refer to this potential redistribution of diagnostic reasoning as “diagnostic outsourcing.” The term does not imply the disappearance of clinical reasoning, nor is it intended to oppose molecular diagnostics. Rather, it describes a conceptual risk associated with increasingly compartmentalized diagnostic pathways, in which biological information may establish disease identity while interpretation of its functional expression becomes a subsequent rather than integrative component of diagnostic reasoning. The clinical relevance of neurophysiology therefore resides not only in signal acquisition or fulfilment of diagnostic criteria, but in integrating electrophysiological findings with anatomical distribution, disease chronology, physiological reserve, compensatory mechanisms, and the clinical phenotype. Professional recommendations similarly emphasize that appropriate electrodiagnostic assessment requires specific expertise and integration of nerve conduction and needle EMG findings within the clinical context [12]. The same electrophysiological abnormality may indicate active dysfunction, recovery, adaptation, or irreversible damage depending on its temporal and clinical context.

### 3.3. Guidelines Preserve Neurophysiology but Underestimate Neurophysiological Expertise

The continued relevance of clinical neurophysiology is reflected in contemporary international guidelines and diagnostic criteria. Electrophysiological investigations remain integral to the diagnostic assessment of several neuromuscular disorders, although their position within diagnostic algorithms varies according to the disease [13,14,15,16,17]. Nerve conduction studies, EMG, repetitive nerve stimulation (RNS), and single-fiber electromyography (SFEMG) remain incorporated into diagnostic pathways, disease classification criteria, and, in selected conditions, therapeutic decision-making and clinical trial eligibility. However, a distinction should be made between recommending or incorporating an electrophysiological investigation within diagnostic criteria and defining the expertise required for its appropriate performance and interpretation. The evolution of diagnostic criteria for immune-mediated neuropathies provides a particularly illustrative example. In chronic inflammatory demyelinating polyradiculoneuropathy (CIDP), the 2021 EAN/PNS guideline assigns electrodiagnostic testing a central rather than merely supportive role: motor and sensory nerve conduction abnormalities form part of the core diagnostic criteria, whereas CSF analysis, imaging, nerve biopsy, and treatment response serve as supportive criteria in selected diagnostic circumstances [15]. Fulfilment of numerical conduction criteria, however, does not eliminate the need to distinguish acquired demyelination from abnormalities related to axonal loss, entrapment neuropathies, technical factors, or other acute and chronic neuropathic processes. Misinterpretation of nerve conduction findings is a recognized contributor to CIDP misdiagnosis and may result in inappropriate immunotherapy [18,19]. Recognition of conduction block, reversible conduction failure, temporal dispersion, and axonal degeneration therefore requires integration of waveform morphology, anatomical distribution, serial examinations, disease chronology, and clinical evolution [18,19]. A similar interpretative challenge occurs in MG, where electrophysiological assessment complements clinical and serological evaluation and becomes particularly relevant when antibody testing is negative or diagnostic uncertainty persists. SFEMG is highly sensitive for detecting impaired neuromuscular transmission, but increased jitter is not specific for MG. It may also occur in the context of denervation and reinnervation in chronic neuropathies and motor neuron disease, as well as in some myopathies. International SFEMG guidelines therefore recommend considering underlying neuropathic or myopathic abnormalities when interpreting increased jitter [14]. The diagnostic value of SFEMG consequently derives not only from its sensitivity, but also from appropriate muscle selection, technical performance, and interpretation within the clinical and conventional electrodiagnostic context [14,20].

ALS provides a further example. The Gold Coast criteria recognize EMG as a means of demonstrating lower motor neuron dysfunction, requiring evidence of both chronic neurogenic change and ongoing denervation within an appropriate anatomical distribution [17,21]. Electrophysiological assessment can therefore identify active denervation, chronic reinnervation, regional dissemination, and clinically unapparent lower motor neuron involvement. These findings require interpretation in relation to the clinical phenotype and potential disease mimics rather than being reduced to a binary concept of “EMG positivity.”

Guidelines are indispensable for standardization, but standardized criteria and expert physiological interpretation serve complementary purposes. The former provides reproducible diagnostic frameworks; the latter determines whether an electrophysiological abnormality is technically valid, pathophysiologically coherent, and clinically relevant in the individual patient. The continued inclusion of neurophysiology in contemporary diagnostic frameworks therefore supports not only the role of electrophysiological testing, but also the importance of expertise in its interpretation.

## 4. Clinical Neurophysiology Across the Spectrum of Neuromuscular Disorders

**Figure 2:**
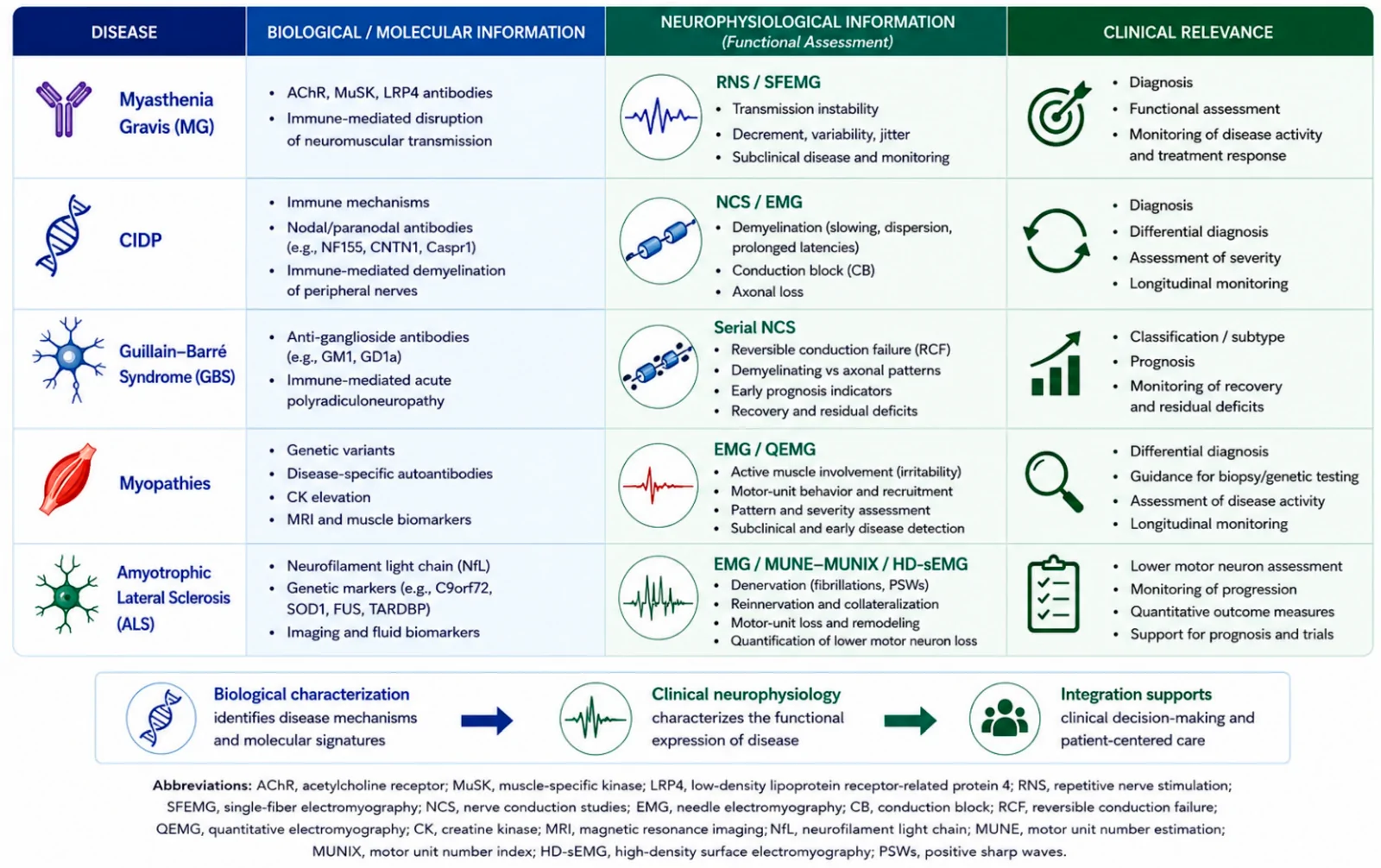
Clinical Neurophysiology Across Major Neuromuscular Diseases. Caption: Comparative overview of the complementary contributions of biological biomarkers and clinical neurophysiology across major neuromuscular disorders. Neurophysiological techniques characterize the functional expression of disease, including neuromuscular transmission failure, conduction abnormalities, muscle dysfunction, denervation, reinnervation, and motor unit loss—providing information that complements molecular, serological, and imaging findings and supports diagnosis, longitudinal assessment, and therapeutic evaluation.

The conceptual framework outlined above acquires its clinical relevance when applied to individual neuromuscular disorders. The following sections examine representative conditions in which neurophysiological assessment complements biological diagnosis by providing information relevant to disease characterization, differential diagnosis, therapeutic decision-making, and longitudinal assessment. Rather than reviewing individual electrophysiological techniques comprehensively, I focus on clinical scenarios that illustrate the contribution—and limitations—of physiological information within contemporary multimodal diagnostic pathways (Figure 2).

### 4.1. MG: From Biological Diagnosis to Functional Assessment

MG (MG) provides a paradigmatic example of the complementary roles of biological and physiological assessment. Antibodies against the acetylcholine receptor (AChR), muscle-specific kinase (MuSK), and low-density lipoprotein receptor-related protein 4 (LRP4) refine disease classification and support targeted therapeutic strategies [22,23,24,25,26,27,28,29], but antibody status does not directly quantify the functional impairment of neuromuscular transmission. SFEMG abnormalities, particularly increased jitter and blocking, may correlate with clinical severity and can provide objective evidence of transmission instability when serological and clinical findings are discordant [30,31].

RNS and SFEMG therefore provide complementary functional information by assessing neuromuscular transmission in vivo [14,20]. RNS demonstrates transmission failure during repeated activation, whereas SFEMG quantifies temporal variability of neuromuscular transmission and may detect instability before clinically apparent weakness. This contribution is especially relevant in seronegative ocular MG, in which negative antibody testing does not exclude the diagnosis and electrophysiological evidence may support the clinical assessment when currently available biological markers remain inconclusive [13,32,33,34,35,36]. Findings must, however, be interpreted according to the clinical phenotype, muscle selection, and technical quality rather than as isolated binary results.

#### RNS in Emergency and Critical Care: Botulism as a Clinical Example

RNS may be particularly valuable in emergency and critical care, where rapidly progressive bulbar weakness or acute respiratory failure requires prompt physiological localization. In suspected myasthenic crisis, RNS can provide objective evidence of impaired neuromuscular transmission while serological results are still pending [37]. Botulism provides an especially informative complementary example because acute presynaptic neuromuscular junction failure may clinically resemble other causes of descending paralysis, whereas electrophysiological testing can demonstrate a characteristic disorder of transmission and thereby contribute to rapid diagnostic orientation [38]. In these settings, neurophysiology is clinically actionable not because it replaces etiological testing, but because it identifies the physiological mechanism at a time when biological characterization may still be incomplete.

### 4.2. CIDP and Guillain–Barré Syndrome: Understanding Conduction Block and Reversible Conduction Failure

Immune-mediated neuropathies provide a particularly informative model of the relationship between biological characterization and functional assessment. In CIDP (CIDP) and Guillain–Barré syndrome (GBS), nerve conduction studies contribute not only to diagnosis and classification but also to the characterization of the mechanisms underlying conduction failure and their evolution over time [16,39]. The biological characterization of these disorders has advanced substantially with the identification of antibodies directed against nodal and paranodal proteins, including neurofascin-155, contactin-1, and Caspr1, contributing to the recognition of autoimmune nodopathies as distinct clinicopathological entities [40,41,42,43,44]. These discoveries have refined disease classification and identified phenotypes with distinct clinical characteristics and treatment responses. Neurophysiological assessment provides complementary information by determining the functional consequences of immune-mediated injury on impulse propagation [41,42]. Nerve conduction studies can distinguish slowing compatible with demyelination, conduction failure with preserved axonal continuity, and secondary axonal degeneration. Importantly, these patterns may evolve during the course of disease, and their distinction has diagnostic, prognostic, and potentially therapeutic implications [45]. This dynamic physiological perspective is particularly relevant to the interpretation of conduction block and reversible conduction failure in CIDP, GBS, and related immune-mediated neuropathies.

#### 4.2.1. Conduction Block: A Physiological Signature of Reversible Dysfunction

Conduction block represents a particularly informative electrophysiological manifestation of impaired impulse propagation in immune-mediated neuropathies. Neurophysiologically, it reflects failure of an action potential to propagate across a focal nerve segment despite preserved distal axonal continuity [46], resulting in a reduction in compound muscle action potential (CMAP) amplitude or area following proximal compared with distal stimulation. The pathophysiological significance of conduction block extends beyond its role as a diagnostic criterion. Depending on the underlying substrate, conduction failure may result from segmental demyelination, disruption of nodal sodium-channel organization, paranodal dysfunction, or other mechanisms that impair saltatory conduction without immediate axonal degeneration [47]. Its recognition therefore identifies functional failure of impulse propagation that may, in appropriate circumstances, remain reversible. Serial electrophysiological assessment is particularly informative in distinguishing recovery of conduction from progression toward axonal loss. Resolution of conduction block with restoration of impulse propagation suggests reversible dysfunction, whereas declining distal CMAP amplitudes may indicate secondary axonal degeneration and a less favorable functional substrate. Thus, similar degrees of clinical weakness may arise from different physiological mechanisms with distinct prognostic and potentially therapeutic implications.

#### 4.2.2. Reversible Conduction Failure: Beyond Demyelination

Reversible conduction failure (RCF) provides a particularly informative example of the interpretative contribution of clinical neurophysiology. Characterized through the work of Uncini and collaborators, RCF challenged the traditional assumption that conduction failure in acute immune-mediated neuropathies necessarily reflects segmental demyelination [48]. Transient conduction failure may instead result from functional disruption of nodal excitability and sodium-channel organization without structural myelin disruption. In these patients, conduction abnormalities may resolve rapidly on serial studies without the temporal evolution or development of temporal dispersion expected in classical demyelination, supporting restoration of nodal function rather than remyelination [49]. This distinction emphasizes the importance of interpreting electrophysiological abnormalities as dynamic rather than exclusively static phenomena. Serial nerve conduction studies can distinguish reversible nodal dysfunction from demyelination and evolving axonal degeneration by documenting the temporal behavior of conduction abnormalities [50]. RCF therefore provides a physiological demonstration that conduction failure may occur in structurally preserved axons and may recover without the electrophysiological features expected from remyelination. This concept is particularly relevant in acute motor axonal neuropathy and related immune-mediated neuropathies, in which nodal dysfunction may produce transient conduction failure and potentially mimic demyelinating conduction block. Recognition of this pattern can therefore influence electrophysiological classification and prevent the erroneous attribution of reversible nodal dysfunction to segmental demyelination [46,51,52,53]. More broadly, RCF illustrates why the interpretation of nerve conduction abnormalities requires consideration of anatomical distribution and, critically, temporal evolution. Similar abnormalities observed at a single time point may arise from different pathophysiological substrates; serial assessment can reveal whether impulse propagation recovers, remains impaired, or progresses toward axonal degeneration. In this setting, neurophysiology provides dynamic functional information that complements biological characterization and contributes to understanding the mechanisms underlying neurological dysfunction.

### 4.3. Myopathies: Electrophysiology Beyond Morphological Classification

The diagnostic landscape of muscle disease has changed substantially with the development of next-generation sequencing, muscle MRI, and molecular pathology. Genetic testing can establish the molecular basis of many inherited myopathies, while MRI provides information on the distribution of muscle involvement, inflammation, fibrosis, and fatty replacement [54,55,56,57]. These approaches have improved disease classification but provide information that is complementary to the physiological assessment of muscle and motor unit function. Needle EMG characterizes motor unit morphology, recruitment, spontaneous activity, and activation patterns, helping to distinguish primary muscle disease from neurogenic disorders and to identify active membrane instability or superimposed neurogenic changes [58]. This distinction remains clinically relevant when weakness has an uncertain origin or when molecular findings, including variants of uncertain significance, require interpretation within the clinical phenotype [59].

In inflammatory myopathies, EMG may provide evidence of active muscle involvement through fibrillation potentials, positive sharp waves, complex repetitive discharges, and myopathic motor unit recruitment [60,61,62]. These findings are not disease-specific and must be interpreted alongside serum biomarkers, autoantibody profiles, imaging, and clinical findings. Nevertheless, electrophysiological evidence of active muscle-fiber irritability may contribute to the assessment of ongoing disease activity when other measures are inconclusive and may support longitudinal evaluation during treatment [63]. Beyond conventional qualitative assessment, quantitative EMG (QEMG) provides objective analysis of motor unit potential characteristics and recruitment patterns. Quantification of parameters such as motor unit potential duration, amplitude, area, complexity, and firing behavior can improve reproducibility and reduce dependence on subjective pattern recognition [64,65].

Modern approaches incorporating automated motor unit potential analysis and signal decomposition further extend the potential of EMG to characterize motor unit remodeling and to generate quantitative measures suitable for longitudinal assessment. However, their role as validated treatment-response biomarkers or clinical-trial endpoints remains less established than their diagnostic application and requires further prospective validation. The development of quantitative electrophysiological biomarkers is particularly relevant as disease-modifying therapies emerge for inherited muscle disorders. In Duchenne muscular dystrophy, for example, electrical impedance myography (EIM) has demonstrated sensitivity to longitudinal disease progression and correlations with established functional measures. Longitudinal studies have also shown changes following corticosteroid initiation, and EIM-derived measures have been proposed as exploratory endpoints capable of reducing sample-size requirements in therapeutic trials [66,67]. These findings illustrate the potential for quantitative physiological measures to complement molecular and functional outcomes in therapeutic studies, although further validation and standardization remain necessary.

Thus, the contemporary contribution of clinical neurophysiology to myopathies extends from differential diagnosis and physiological characterization to the development of quantitative measures of disease evolution. Genetics defines molecular substrate, imaging characterizes anatomical involvement, and neurophysiological techniques provide complementary information on muscle and motor unit function. Their integration may become increasingly relevant for longitudinal monitoring and therapeutic studies as precision treatments continue to expand.

### 4.4. ALS: The Physiology of Motor Neuron Degeneration

**Figure 3:**
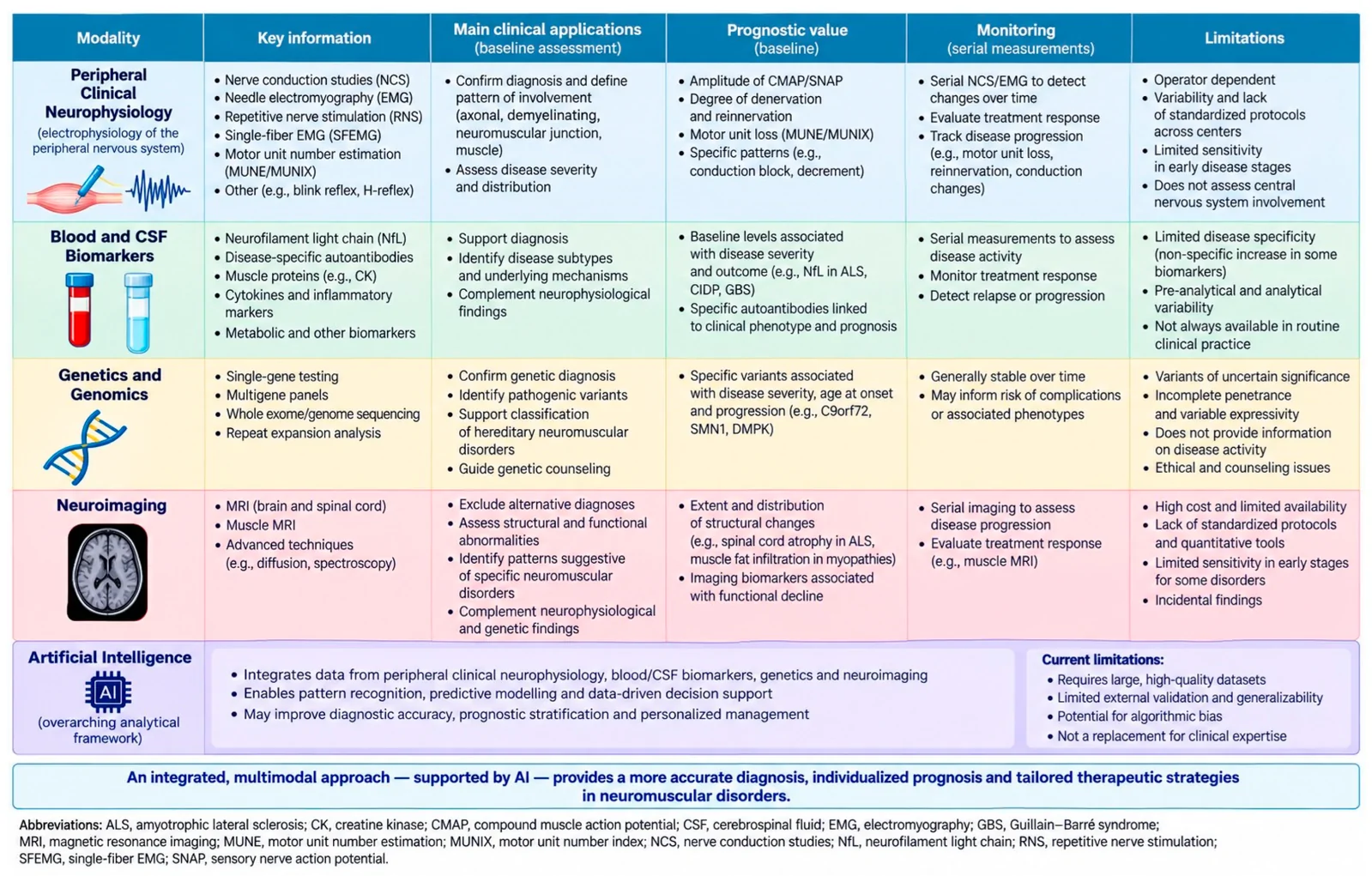
Complementary contributions of multimodal assessment in neuromuscular disorders. The table summarizes the complementary information provided by biological biomarkers, imaging, and peripheral clinical neurophysiology, highlighting their principal strengths, limitations, and potential clinical applications. Peripheral clinical neurophysiology provides direct functional assessment and may contribute to diagnosis and prognostic stratification at baseline, while longitudinal monitoring requires serial measurements. Artificial intelligence (AI) is represented as an overarching analytical framework capable of processing and integrating data across modalities rather than as an independent diagnostic modality.

ALS (ALS) illustrates the complementary information provided by biological and physiological biomarkers [68,69]. Over the last decade, fluid biomarkers—particularly neurofilament light chain (NfL)—together with advances in molecular genetics and neuroimaging have substantially improved disease characterization, prognostic assessment, and patient stratification for clinical trials [70,71,72,73]. NfL is an objective marker of neuroaxonal injury and, in ALS, predominantly reflects upper motor neuron axonal degeneration; it does not directly characterize the functional status, loss, or compensatory remodeling of individual lower motor units. EMG addresses this complementary physiological dimension by documenting active denervation, chronic reinnervation, motor unit instability, and reduced recruitment [17,74,75]. These findings reflect the dynamic interaction between motor neuron degeneration and compensatory collateral reinnervation and may reveal lower motor neuron involvement that is not yet clinically apparent. Contemporary diagnostic frameworks, including the Awaji recommendations and Gold Coast criteria, recognize electrophysiological evidence as a means of demonstrating lower motor neuron dysfunction [21,76,77,78,79]. Fasciculation potential deserves particular attention: in the appropriate clinical and electrophysiological context, their presence can provide evidence of active lower motor neuron dysfunction, especially when accompanied by chronic neurogenic motor unit changes. Their diagnostic value depends on distribution, morphology, associated denervation/reinnervation findings, and the exclusion of benign or other neurogenic causes rather than on their mere presence. Beyond diagnosis, the burden and evolution of fasciculation activity may provide information about ongoing motor unit instability and disease progression, although its prognostic interpretation requires standardized acquisition and longitudinal validation [79,80]. Emerging quantitative neurophysiological techniques further extend this functional assessment. Motor unit number estimation (MUNE) and motor unit number index (MUNIX) provide quantitative measures of motor unit loss, while high-density surface EMG enables increasingly detailed characterization of motor unit recruitment, discharge properties, and reinnervation dynamics [81,82,83,84]. These approaches complement conventional EMG by providing reproducible physiological measures suitable for longitudinal assessment and, potentially, therapeutic trials. Integrating neurophysiological and fluid biomarkers may therefore provide a more complete characterization of ALS progression: NfL captures neuroaxonal injury, whereas electrophysiological measures characterize lower motor unit loss, dysfunction, and compensatory remodeling. This distinction is particularly relevant in therapeutic studies, where biological target engagement does not necessarily imply preservation of motor unit function (Figure 3) [85].

## 5. Artificial Intelligence and Clinical Neurophysiology: From Pattern Recognition to Physiological Reasoning

Artificial intelligence (AI) is increasingly influencing clinical neurophysiology through machine-learning and deep-learning approaches capable of analysing complex electrophysiological signals rapidly and quantitatively. Applications include automated waveform classification, quantitative EMG, motor unit decomposition, high-density surface electromyography, electroencephalography, and multimodal signal processing [86,87]. In electrodiagnostic and neuromuscular medicine, AI-based models have shown promising performance in distinguishing normal, neurogenic, and myopathic signals and in extracting quantitative features that may reduce interobserver variability [88]. However, promising analytical performance should not be equated with clinical readiness. Current AI models in neurophysiology face important limitations related to dataset size and representativeness, heterogeneous acquisition protocols, annotation quality, overfitting, limited external validation, and differences among laboratories, equipment, and patient populations [89]. A recent review of AI-based needle EMG classification found that many studies relied on small or repeatedly reused datasets and were affected by methodological bias or overtraining, concluding that current models are not yet sufficiently validated for routine clinical implementation [90]. More broadly, models trained on selected datasets may perform substantially less well when applied to heterogeneous real-world populations, emphasizing the need for prospective and external validation. A further limitation concerns overreliance on pattern recognition. Algorithms can identify statistical regularities within electrophysiological signals, but similar waveform abnormalities may arise from different pathophysiological processes. Classification of a motor unit potential as “neurogenic” or “myopathic,” for example, does not by itself establish disease etiology, anatomical distribution, temporal evolution, or clinical relevance. Moreover, apparently high classification accuracy may partly reflect dataset characteristics rather than robust physiological discrimination; methodological studies have shown substantially lower performance when more rigorous validation strategies are applied. Overconfidence in algorithmic output may therefore introduce automation bias, particularly when AI-generated classifications are accepted without adequate consideration of signal quality, technical artefacts, atypical phenotypes, or discordant clinical findings [91].

These limitations do not diminish the potential of AI but help define the tasks for which automation is most appropriate. Automated measurements, waveform detection and classification, signal quality control, motor unit decomposition, quantitative feature extraction, and prioritization of large datasets are particularly suitable for computational assistance. By contrast, determining whether an electrophysiological abnormality represents active disease, recovery, chronic compensation, technical artefact, or an alternative pathological process requires integration with neurological examination, anatomical localization, disease chronology, biological biomarkers, treatment history, and the evolving clinical phenotype [92,93,94,95,96,97]. The most realistic model is therefore one of augmented neurophysiology, in which AI performs reproducible computational tasks while the neurophysiologist retains responsibility for physiological interpretation and clinical integration. This approach is consistent with the recent International Federation of Clinical Neurophysiology (IFCN) position statement, which emphasizes that AI should support rather than replace clinical expertise and highlights the need for diverse datasets, transparency, rigorous validation, patient safety, and appropriate expert oversight. Successful implementation will also require attention to workflow integration, interoperability, regulatory oversight, data governance, clinician training, accountability, and the risk of automation bias. AI may ultimately facilitate multimodal models integrating electrophysiological recordings with imaging, biological biomarkers, wearable sensors, and other digital health data [98,99,100,101]. Such integration could expand the amount and complexity of physiological information available for clinical decision-making, but its value will depend on prospective validation and demonstration of clinical utility rather than analytical performance alone. In this framework, AI is best regarded not as an alternative to physiological reasoning, but as a computational tool capable of extending its quantitative and integrative potential [102,103].

## 6. Limitations of the Current Review

This review has several limitations that should be acknowledged. First, it is a critical narrative review rather than a systematic review or meta-analysis; therefore, the literature discussed was selected to illustrate representative conceptual and clinical examples rather than through a predefined systematic search strategy. Consequently, relevant studies may not have been included, and the evidence presented should not be interpreted as an exhaustive assessment of the available literature.

Second, the review focuses on selected neuromuscular disorders—MG, immune-mediated neuropathies, myopathies, and ALS—chosen because they provide representative examples of the interaction between biological and physiological information. The proposed framework may therefore not be directly generalizable to the full spectrum of neuromuscular diseases or to other areas of clinical neurophysiology. Third, neurophysiological techniques themselves have important limitations. Their diagnostic performance depends on technical quality, appropriate study design, operator expertise, disease stage, anatomical distribution, and correct interpretation within the clinical context. Electrophysiological abnormalities are not invariably disease-specific, and normal findings do not necessarily exclude disease, particularly in early, focal, or intermittently expressed disorders.

Finally, several emerging approaches discussed in this review—including quantitative EMG, MUNE/MUNIX, high-density surface EMG, and AI-assisted signal analysis—remain incompletely standardized across centers, and their roles as longitudinal biomarkers or therapeutic trial endpoints require further prospective and external validation. The conceptual model proposed here should therefore be regarded as a framework for integrating physiological information with molecular, imaging, and computational biomarkers rather than as a replacement for established diagnostic algorithms.

## 7. Conclusions and Future Perspectives

The evolution of neuromuscular medicine increasingly requires integration rather than competition between biological and physiological approaches. Molecular biomarkers, genetics, imaging, and emerging computational tools provide increasingly precise characterization of disease mechanisms, while clinical neurophysiology contributes direct assessment of their functional expression. The examples discussed in this review illustrate how these complementary sources of information can refine diagnosis, characterize disease activity and progression, and support therapeutic decision-making.

The future of clinical neurophysiology will depend on its ability to evolve within this multimodal framework. Quantitative EMG, motor unit analysis, high-density recordings, digital biomarkers, and AI-assisted signal processing offer opportunities to develop more objective and reproducible physiological measures. Future research should prioritize prospective multicenter validation, methodological standardization, and the integration of neurophysiological measures with molecular, imaging, and clinical outcomes, particularly for longitudinal monitoring and therapeutic trials.

This evolution also has implications for training. Increasing automation of signal acquisition and analysis should be accompanied by continued emphasis on physiological reasoning, technical quality, recognition of artefacts, and integration of electrophysiological findings with the clinical phenotype. AI may increasingly support quantitative and pattern-recognition tasks, but its clinical value will depend on appropriate validation and expert oversight.

Clinical neurophysiology should therefore be considered an evolving component of precision neuromuscular medicine rather than a diagnostic discipline to be preserved unchanged. Its future relevance will depend on the capacity to combine rigorous physiological interpretation with emerging biological and computational tools. Such integration may allow precision medicine to characterize not only the molecular identity of disease, but also its functional expression in the individual patient.

## Data Availability

No new data were created or analyzed in this study. Data sharing is not applicable to this article.
